# A covert authentication and security solution for GMOs

**DOI:** 10.1186/s12859-016-1256-6

**Published:** 2016-09-21

**Authors:** Siguna Mueller, Farhad Jafari, Don Roth

**Affiliations:** 1 Department of Molecular Biology, University of Wyoming, 1000 E. University Ave, Laramie, WY 82071 USA; 2 Department of Mathematics, University of Wyoming, 1000 E. University Ave, Laramie, WY 82071 USA

**Keywords:** GMO security, Limits of watermarking, Zero knowledge proofs, Verifiable encryption for GMO

## Abstract

**Background:**

Proliferation and expansion of security risks necessitates new measures to ensure authenticity and validation of GMOs. Watermarking and other cryptographic methods are available which conceal and recover the original signature, but in the process reveal the authentication information. In many scenarios watermarking and standard cryptographic methods are necessary but not sufficient and new, more advanced, cryptographic protocols are necessary.

**Results:**

Herein, we present a new crypto protocol, that is applicable in broader settings, and embeds the authentication string indistinguishably from a random element in the signature space and the string is verified or denied without disclosing the actual signature. Results show that in a nucleotide string of 1000, the algorithm gives a correlation of 0.98 or higher between the distribution of the codon and that of *E. coli*, making the signature virtually invisible.

**Conclusions:**

This algorithm may be used to securely authenticate and validate GMOs without disclosing the actual signature. While this protocol uses watermarking, its novelty is in use of more complex cryptographic techniques based on zero knowledge proofs to encode information.

## Background

The dramatically increasing worldwide utilization of genetically modified plants, animals and microbes (GMOs) presents challenges to ensure security, authenticity and validation of material goods and legal agreements. Similarly to the evolution witnessed in internet protocols, strategic focus is required to anticipate, track and address potential infringements of GMO security. It is imperative that unimpeachable protocols assure product ownership, provide data to track the product supply chain, and to preempt malicious attacks especially related to bioagents, such as weaponized anthrax spores. As GMOs are not tamper proof or invulnerable to outside attack, it is necessary to encode and embed cyber-security data within the GMO genome. An ideal GMO based security mechanism should provide a secure authentication process accessible to relevant parties without revealing the specific signature components to outside parties. Watermarking has been used extensively to establish authentication signatures that validate ownership by providing a mechanism to conceal and recover the required data necessary to authenticate the identification signature of the originator [1–4]. However, in watermarking applications, the identity of the authentication information is disclosed as validity is verified [5–11]. Although these methods would be useful under many scenarios, they are unacceptable in the context of sophisticated GMO security because they would fail under concerted attack based on malicious transfer and signature duplication. For example, Clelland et al. [5], although establishing significant message secrecy, does not protect the key decoding signatures after access by a third party. It also appears that their approach would be best suited to small message concealment because the DNA based message length must be similar to that of the background sonicated genomic DNA (ca 50-150 nucleotides). Ideally, the signature should not be transferrable, and should remain concealed even from third parties utilizing the cryptographic code. Integrating this sophisticated cryptographic modification is critical for sustained next generation security of GMOs. A focused adversary should only be able to speculate whether a given sequence of nucleotides is a random sequence or a valid signature sequence. Considering a scenario where an attacker might seek to modify a GMO or weaponized bioagent in order to adversely affect function, coercion of individuals having information components or even alignment of whole genome sequences from target and wild type stains via BLAST or other programs should have an extremely low probability of identifying and/or validating an authentic signature. Clearly any security approach also must align with prokaryotic and eukaryotic GMO development and application technologies.

In this paper we demonstrate a novel algorithm that addresses these concerns using advanced cryptographic techniques. For every signature string generated, the developer generates a populating subset with fake signatures (a random sequence of nucleotides of the same length as a valid signature). Thus, each clone would contain adequate information for identification whether or not it is a signature carrying clone. The valid signature can be established by techniques developed in [12] and would be available to the developer and/or third party inspector. Those techniques assure that the input information is correctly received on the output side, i.e. when they are stored and read out there is no loss or change of information, while there is no concern that someone would actively modify or manipulate the data. Since each clone contains a valid or fake signature element, it would be virtually impossible to correctly select the authentic signature. We emphasize that while our algorithm uses watermarking, it is not a watermarking protocol per se. The process of watermarking by itself does not provide adequate security during its verification as it allows potentially malicious transfer and signature duplication. Our protocol uses sophisticated novel cryptanalytic attack models and protection mechanisms. While many of the existing methods use classic cryptography such as symmetric - AES, nonsymmetric -RSA and more classical approaches, we use zero-knowledge and confirmer signature techniques applied to GMOs. These are much more advanced crypto systems than AES and RSA. This algorithm should become the standard in implementation of this technology in practice.

## Comparison with other work

### Practical realization and combination with data-encoding mechanisms into DNA

Embedding of data in DNA has received a lot of attention. Previous algorithm proposals primarily concerned about biological aspects and correct and efficient decoding. Heider and Barnekow [13, 14] focus on error detection and correcting properties - not in the sense of cryptography - but inside the genome, to detect and correct mutations occurring during cell division that might destroy the information that is ’encrypted’ (i.e., hidden), inside the genome. As such the DNA medium can be interpreted as a noisy channel and has been addressed by tools of digital coding and information theory [11]. Depending on usage requirements in living cells, our watermarking step might benefit from such additional features and could easily be combined with theirs or related algorithms. Yachie et al. [15] considered error detection and correction of the data-encoded DNA sequence inside of living organisms. Their approach is a refined repetition code that avoids multiple segments of the same DNA sequence within a single genome. Our method can be combined with their alignment-based DNA-data storage and retrieval method, or any of the sequence alignment methods. In fact, we propose a modern alignment method with provably secure decoding properties in [12].

However, no attention has been placed on the cryptographic aspect of the problem. In particular, in the case of ownership watermarks, biocompatibility along with the correct and efficient encoding is not enough. The embedded information that is stored and retrieved additionally requires specific cryptographic security requirements unique to this situation. It is imperative that the secret key remains hidden during watermark verification to prevent the unintended copying of signature data or specified information. These are independent cryptographic security considerations established in this paper.

### Clarification about utilization of cryptography

In contrast to correctness of encoding and decoding and efficiency, cryptography considers the security aspects of a (digital) communication medium. These security concerns are unique to the DNA setting. An example can be seen work by Gibson et al. [16] who established the synthesis and assembly of a synthetically designed bacterial cell. Watermark sequences are included which distinguish the synthetically designed from naturally occurring DNA and cells. This type of watermark does not yield unique ownership in that watermark verification is only done by multiplex PCR and the entire watermarking procedure could be imitated by others. They were not concerned about cryptographic security features, e.g., if someone were to produce a harmful bacterial clone carrying their watermark information. How would they refute this clone was not theirs?

Haughton and Balado [11] first incorporated a secret *k* to keep the encoding secret. The key is shared only between an encoder and decoder. This has the advantage that only the encoder and decoder will have knowledge of the secret message that is embedded in DNA. In the context of ownership watermarking, unfortunately, this scenario is not fully satisfactory. It requires that verification of the watermark is only possible to a selected list of decoders which has to be determined prior to embedding the watermark. Once the watermark is placed, the watermark verification process is only possible within this fixed set of users. In the case of verification of a watermark ownership to a user outside of this fixed set, this scenario is not applicable.

Heider and Barnekow [13] suggest to integrate several private and public key cryptographic algorithms, by employing encryption or a one-time pad. Both are done to create a short binary message. Although the authors did not make this explicit in their work, the first obvious advantage of this approach is that by doing so the information to be hidden inside the DNA is now scrambled inside a binary string. However, it is imperative to note that they do not utilize any of the mentioned cryptographic algorithms. What is needed for DNA-Crypt is a plaintext message (the information to be hidden inside the DNA) that is efficiently converted to a binary string. Thus, they correctly argue, that any function, mapping, or algorithm, which takes meaningful input and coverts it to binary, can be used for their purpose. Their main concern is only the output binary string. They do not incorporate any cryptographic features. They do not consider security, cryptographic approaches, or utilize encryption and decryption. In fact, they argue that the keys used for these cryptographic algorithms could be exchanged with other users. However, precisely for private key crypto, keeping the keys secret is the most important requirement to ensure security. Clearly, their concern is not to utilize the mentioned algorithms for their cryptographic features, but mainly to generate a binary string. Their concern is for better storage utilization, and hence, the cryptographic integration is only for compression purposes of text data into binary (source compression into binary). In summary, all that is utilized by DNA-Crypt is a binary encoding table [14].

In summary, while data embedding methods have benefited from numerous disciplines of digital communication theory, unique requirements of cryptography and security requirements are first addressed in this work. It is crucial to note, that our work can seamlessly be combined with previous data embedding methods. Balado-Haughton [17] determine the maximal number of ways that DNA watermarking can be done, by considering it as a special data hiding problem. Their basic requirement is the primary structure preservation achieved via the redundancy of the genetic code. This does not lead to a unique solution. Depending on biocompatibility constraints and other practical considerations, the tagging of DNA can be performed in various ways. We have not focused on length requirements of the signature sequence, how easily the signatures can be inserted and read, as our method can easily be combined with any others that focus on such issues.

Our work complements these proven practical realizations, can easily be combined with related successful in vivo experiments to hide the secret information in non-coding regions, and addresses the missing security issues not considered before.

Each of [11, 13–16] use some watermarking as a means to ensure tracking or ownership of DNA or organism. Table 1 gives a comparison with our work.

**Table 1:** Comparison with other work

Feature	[11]	[13]	[14]	[15]	[16]	Our method
Added security features in addition to the sequences	yes	no	no	n/a	no	yes
Source compression (via any cryptographic encoding, substitution cipher, or coding theory)	yes	yes	yes	n/a	n/a	yes
Explicit formulation and identification of broader security requirements and goals	no	no	no	n/a	no	yes
Adaptation and incorporation of novel cryptographic techniques to ensure that sensitive information remains concealed during the verification process	no	no	no	no	no	yes

## Methods

The basic cryptographic building block is defined as a zero-knowledge (ZK) proof of knowledge [18] of a hidden signature to constitute the designated confirmer signature [19]. ZK proofs are both convincing and yet yield no identifying key code information beyond the validity of the assertion being proved. They are typically used to force malicious parties to behave according to a predetermined protocol.

The specific cyber-security protocol is as follows: Let *Σ* be a digital signature scheme given by its key generation protocol *Σ*.keygen which generates a key pair *Σ*.sk and *Σ*.pk consisting of the secret and private key for the signature generation and verification protocols, respectively. Let *Γ* be a cryptosystem described by *Γ*.keygen that generates the pair (public key =*Γ*.pk, private key =*Γ*.sk) to be used for encryption and decryption. Classes and properties required for *Σ* and *Γ* suitable for designated confirmer signatures have been described and analyzed in [19, 20]. To give a specific example, *Σ* will be represented by a suitable *RSA* signature scheme and *Γ* by ElGamal.

The Full Domain Hash RSA [21] signature scheme *Σ* is given by the key pair (*Σ*.pk=(*N*,*e*), *Σ*.sk=*d*) where *N* is an RSA modulus and *ed*≡1 mod *ϕ*(*N*). The keys here are those used by the signer. With all computations in $\mathbf {Z}_{N}^{*}$, a valid signature *σ* on a message *m* is defined via *σ*=*H*(*m*)^d^, where *H* is a public hash function. The verification equation will make use of the following one-way function and image

1 $$ f(x) = x^{e} \text{and}~I = H(m).  $$

Importantly, *f* is homomorphic as for all $x,y \in \mathbf {Z}_{N}^{*}$, *f*(*xy*)=*f*(*x*)*f*(*y*).

For the encryption scheme *Γ* we use ElGamal’s encyption [22]. It operates in a group $({\mathcal {G}}, \cdot)=<g>$ of large enough order where computing discrete logarithms to base *g* is difficult. The confirmer’s secret key is *Γ*.sk=*x* and the corresponding public key is *Γ*.pk=*y*=*g*^*x*^. To encrypt a message $m \in {\mathcal {G}}$, one chooses a random *r* and computes the ciphertext as the pair ${\mathcal {E}}(m)=(g^{r}, m \cdot y^{r}). $ To decrypt a ciphertext (*K*_1_,*K*_2_), one first obtains the session key $k={K_{1}^{x}}=y^{r}$ and then computes *m* as *K*_2_·*k*^−1^.

Let ∘, the binary operation defined on ${\mathcal {G}} \times {\mathcal {G}}$, be the term-wise product (*a*,*b*)∘(*c*,*d*)=(*ac*,*bd*). Fundamental for the construction is the fact that ElGamal is homomorphic since [20],

2 $$ \begin{aligned} {}{\mathcal{E}}(m) \circ {\mathcal{E}}(m') &= (g^{r}, m \cdot y^{r}) \circ (g^{s}, m^{\prime} \cdot y^{s}) \\ &= (g^{r+s}, mm^{\prime} \cdot y^{r+s})= {\mathcal{E}}(m m^{\prime}). \end{aligned}  $$

The space of signatures produced by *Σ* must be the same as the space of messages encrypted by *Γ*. This can be done as follows: the signer chooses two sufficiently large primes *p* and *q* such that *p*^′^=(*p*−1)/2 and *q*^′^=(*q*−1)/2 are prime. The signer sets *N*=*pq* and chooses $g \in \mathbf {Z}_{N}^{*}$ such that (with overwhelming probability) $Q_{N}=\{a^{2}: \, a \, \in \mathbf {Z}_{N}^{*}\} \subseteq < g > \subseteq \mathbf {Z}_{N}^{*}$ and sets ${\mathcal {G}}=Q_{N}$. The signatures produced by *Σ* are mapped into ${\mathcal {G}}$ by squaring all the parameters (even the bases) before performing any modular operations with them. We also assume that the respective keys are verified with a certificate authority and the respective public parameters are publicly accessible. The symbol || will denote the operation which when applied to two strings *m* and *z* results in the ‘usual’ concatenation of the string *m*, and the string *z*.

The individual steps of our cryptographic protocol are described next. Let the given message *m* be the signature data to be signed. Throughout, if *m* is given in its binary representation (quartic representation as DNA), then after appropriate parsing *m*∈**Z**_*N*_ ($m \in {\mathcal {G}}$) is considered to be the representation of the integer *m* modulo *N* (in ${\mathcal {G}}$). The signer first generates a verifiable signature *μ* on *m* using the following steps:

1. The signer chooses $r {R \atop \longleftarrow } \mathbf {Z}_{p^{\prime }q^{\prime }}$ and computes *z*←*g*^*r*^ in $\mathcal {G}$.
2. The signer uses his secret key *Σ*.sk=*d* of RSA-FDH to compute *σ*=*H*(*m*||*z*)^2*d*^.
3. *σ* is converted into DNA bases via our watermarking protocol below and hidden inside the GMO.
4. The signer encrypts *σ* via ElGamal with the confirmer’s public key *Γ*.pk=*y*=*g*^*x*^ and the random *r*, $ {\mathcal {E}}(\sigma) = (K_{1},K_{2})= (g^{r}, \sigma \cdot y^{r})$.
5. *μ*=(*K*_1_,*K*_2_) is stored in an electronic database as the designated confirmer signature of *m*.

A candidate signature *μ* of *m* from a public database can only be validated by the TTP according to the following verification protocol.

1. Given *m* and *μ*=(*K*_1_,*K*_2_), the confirmer computes *σ* as the decryption of the ElGamal ciphertext (*K*_1_=*z*,*K*_2_) using the secret key *x*.
2. The confirmer verifies if *σ* is a valid RSA-FDH signature of *m*||*z* by testing *σ*^*e*^=*H*(*m*||*z*)^2^ using the signer’s public key e.
3. The signature *μ* is accepted as valid if and only if this verification step passes.

Therefore, the algorithm runs as follows:

1. Determine the number of occurrences *N*_*i*_ of each codon *C*_*i*_ in the host genome, see e.g. [23].
2. Determine the number *M*_*j*_ of binary triplets *B*_*j*_ in the given binary sequence (with filling in of mock elements to yield a number of characters divisible by 3).
3. Let ${\mathcal {N}}=\{A,C,G,T\}$ be the set of nucleotides. There are 24 ways in which these can be ordered. Let *n*_1_,*n*_2_ be the first two nucleotides, and *n*_3_,*n*_4_ the latter two in an arbitrary ordering.
4. Associate with each triplets *B*_*j*_ of a given binary string a set of possible codons *C*_*j*_, *B*_1_↦{*n*_1_*n*_1_*n*_1_, *n*_1_*n*_1_*n*_2_, *n*_1_*n*_2_*n*_1_,…,*n*_2_*n*_2_*n*_2_},…*B*_8_↦{*n*_3_*n*_3_*n*_3_, *n*_3_*n*_3_*n*_4_, *n*_3_*n*_4_*n*_3_,…,*n*_4_*n*_4_*n*_4_}, where the associated codon list excludes the ATG start codon [11].
5. The number of times each text triplet is represented by each associated codon *C*_*j*_ is determined according to the following: For each *B*_*j*_ determine the number of occurrences *N*_*j*_ of each of the eight codons *C*_*j*_ that is associated to *B*_*j*_ via the above mapping. Spread out the *N*_*j*_ occurrences of each *B*_*j*_ according to match the individual numbers of occurrences of their associated codons *C*_*j*_.

## Results and discussion

The main difference between confirmer signatures and ordinary electronic signatures based on watermarking techniques is that confirmer signatures are not self-authenticating. Our algorithm is essentially based on the confirmer signature concept. In this case, the entity requiring proof of authentication (the “verifer”) cannot check the ownership or the validity of a signature unless a legitimate party confirms or disavows it [24, 25]. While the original signer (the originating product owner, developer or a delegate) can confirm the signature, it also may be verified by a semi-trusted third party (TTP) “confirmer” [26]. There are several advantages to limiting signature validation access to the confirmer and verifier. First, it protects the signer against coercion. Second, it protects the buyer if the signer becomes unavailable. Third, it validates the authentic signature without disclosing the actual signature sequence to an adversary who may even masquerade as a verifier. While the constant involvement of a TTP in the cryptographic protocol gives more power to the TTP, it also enables the TTP to obtain and disclose the signature in case of dispute and has additional important forensic implications. Following successful protocol implementation, the verifier and TTP have certifiable validation that the GMO contains a specific identifying signature although the verifier never acquires the exact signature [20].

**Fig. 1:**
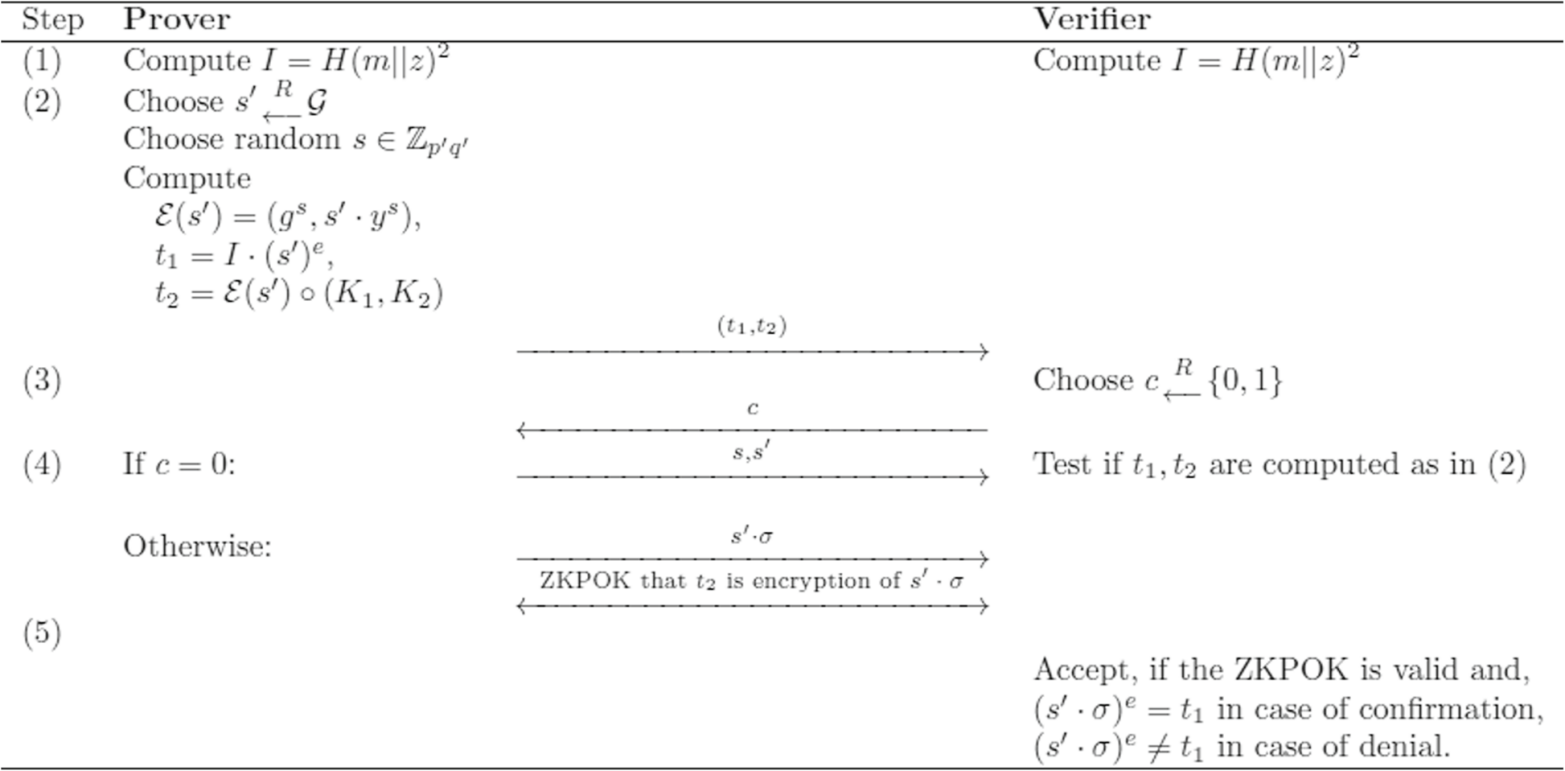
ZK proof of knowledge to verify authenticity. The prover and verifier are given the public input, an alleged signature (*K*_1_,*K*_2_)=*μ* with *z*=*K*_1_, and the message (signature data) *m*. If $\mu ={\mathcal {E}}(\sigma)$ is generated as above, then *t*_1_=(*s* ^′^·*σ*)^*e*^ and *t*_2_=(*g* ^*s*+*r*^,*s* ^′^ *σ*·*y* ^*s*+*r*^) where *s* is the randomness used in ElGamal to encrypt *s* ^′^, and *r* is that used to encrypt *σ*. In this case, the protocol allows the prover to confirm the signature in ZK. If *μ* is a falsely implied signature, the protocol allows the prover to deny the signature in ZK

**Fig. 2:**
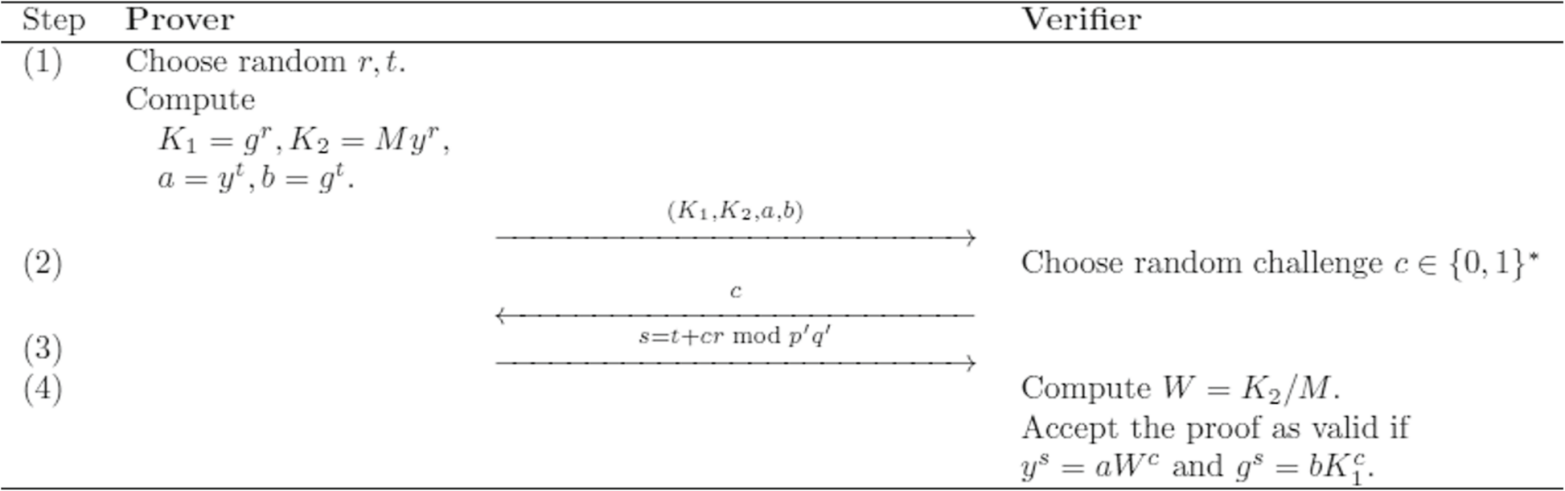
Proof that (*K*_1_,*K*_2_) is the encryption of the given message *M* under ElGamal. If the prover can successfully answer two distinct challenges *c*_1_,*c*_2_ with two acceptable answers *s*_1_,*s*_2_ then the verification step results in $ y^{s_{1}-s_{2}} = W^{c_{1}-c_{2}} \text {and} g^{s_{1}-s_{2}} = K_{1}^{c_{1}-c_{2}}$ (see [27]). Thus, if *c*_1_−*c*_2_<*p* ^′^ *q* ^′^ a value *r* exists such that *r*=log_*y*_ *W*=log_*g*_ *K*_1_=(*s*_1_−*s*_2_)/(*c*_1_−*c*_2_) mod *p* ^′^ *q* ^′^. Consequently, *W*=*y* ^*r*^, *K*_1_=*g* ^*r*^, and *K*_2_=*M* *y* ^*r*^. This proves that (*K*_1_,*K*_2_) is indeed an encryption of the message *M* under ElGamal that can be translated into a ZKPOK according to [20]

Specifically, the confirmer and/or signer provides a ZK proof to demonstrate the validity of this signature to the verifier (Fig. 1). A valid signature is not accepted without the confirmation protocol, and a falsely alleged signature can only be repudiated via the denial protocol. In Figs. 1 and 2, the prover is either the confirmer of the signature who can undo encryption via ElGamal with the knowledge of the private key, or the signer who wishes to confirm the validity of signature *μ*. Thus, signature verification can be established by the signer without the involvement of the TTP. The TTP has the ability to undo ElGamal encryption and is the only party who can obtain the signature *σ*. Signature verification is therefore solely based on the encrypted signature $\mu ={\mathcal {E}}(\sigma)$, not the signature *σ* itself. In case of dispute the TTP can make *σ* public, convert it into nucleotides, and determine presence in the GMO.

Step 4 in Fig. 1 requires a protocol for proving that a given ciphertext under ElGamal decrypts to a given message *M*. The general building block is termed the proof of equality of two discrete logarithms [27, 28]. Adapted to our context, this protocol runs as depicted in Fig. 2. This protocol can be modified into a ZK proof of knowledge (ZKPOK) protocol by augmenting the Fig. 2 process by steps (2)–(4) of Fig. 1 [20].

It is important to note that a signature cannot be verified solely from identification of a unique sequence string. With our algorithm, as opposed to standard watermarking methods, a signature is not accepted as valid without the cooperation of the prover or delegate through the cryptographic confirmation protocol. Hence, even if an attacker detects a candidate signature, its validity is known only to the legitimate verifier who interacts with the prover in the protocol. Without the prover, no party can determine whether *σ* is a valid signature for *m* or not. Similarly, the specific denial protocol process ensures that a certain string cannot be denied by the original signer as an invalid signature. The protocol provides objective certifiable authentication of ownership as the signature is retrievable and verifiable by designated parties, e.g. the TTP. The TTP may disclose the signature for verification by other parties although normally, the company’s signature data remain completely hidden. The ZK property ensures that no one has access to the signer’s secret key or the signature. Even if the buyer or a masquerading adversary analyzes via genome sequencing individual GMO’s in a population, the secret key or the signature that allows them to impersonate the verifier cannot be discovered.

**Fig. 3:**
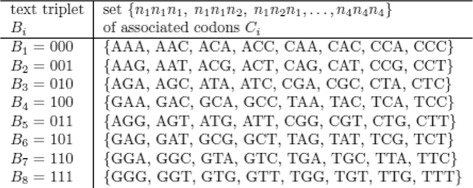
Correspondence between the binary text triplets *B*_*i*_ and DNA codons *C*_*i*_ for the specific example where *n*_1_=*A*,*n*_2_=*C*,*n*_3_=*G*,*n*_4_=*T*. Each of the text triplets is distributed over the associated codon triplets so that the resulting representation resembles the codon bias of the host genome. To demonstrate the watermarking protocol, assume there are 44 occurrences of 000 in the binary text and that the codon frequency values as determined from the entire codon frequency distribution, are: *A* *A* *A*,3.3 *%*, *A* *A* *C*,2.1 *%*, *A* *C* *A*,0.8 *%*, *A* *C* *C*,2.3 *%*, *C* *A* *A*,1.5 *%*, *C* *A* *C*,0.9 *%*, *C* *C* *A*,0.8 *%*, *C* *C* *C*,0.6 *%*, covering a total of 12.5 *%* of the total codon distribution. Among the codons assigned to 000, there are 100·3.3/12.5=26.6 *%* for *AAA*, 17.1 *%* for *AAC* etc. Consequently, we assign (26.6·44)/100∼12 occurrences of 000 to *AAA*, 8 to *AAC*, 3 to *ACA*, 8 to *ACC*, 5 to *CAA*, 3 to *CAC*, 3 to *CCA*, and 2 to *CCC*, covering the total 44 occurrences of 000

The protocol ensures that the signature string is indistinguishable in the electronic signature space as represented by integers [20, 29]. In conjunction with the signature protocol we developed a watermarking algorithm that is designed to provide signature invisibility. The protocol consists of converting the cryptographic signature *σ* into the DNA alphabet such that it is indistinguishable from the endogenous DNA after insertion in the genome. The algorithm effectively camouflages the required authentication and/or tracking data to ensure that an adversary cannot identify the signature as a security or watermarking feature. The process also is reversible. From the nucleotide sequence, the signature can be translated to cryptographic code. The algorithm encodes binary triplets based on the frequency of each codon as determined by the codon bias of the host. Codon bias refers to each organism’s inherent preferences of certain triplet nucleotides for translation into corresponding amino acids. Our approach is solely DNA based and does not require transcription or translation of sequences. Alignment of the message with the host codon bias is designed to better hide the message in the genomic background. Each binary bit is assigned to a choice of two specific nucleotides *n*_*i*_∈{*A*,*C*,*G*,*T*}, i.e. 0↦*n*_1_or *n*_2_, 1↦*n*_3_or *n*_4_ to mirror the codon frequencies of the host with the frequencies of the binary text triplets. Figure 3 shows the correspondence between the binary text triplets *B*_*i*_ and DNA codons *C*_*i*_ for the specific example where *n*_1_=*A*,*n*_2_=*C*,*n*_3_=*G*,*n*_4_=*T*. Each of the text triplets is distributed over the associated codon triplets so that the resulting representation resembles the codon bias of the host genome. Further improvement can be made by renaming and reordering, as the choice of the *n*_*i*_ is arbitrary. Renaming the codons by matching the obtained string with the host frequency distribution results in a correlation of the obtained with the host frequency distribution of typically 0.98 or more.

**Fig. 4:**
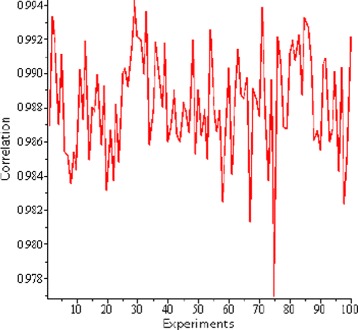
Representation of the correlation between codon frequency distribution produced via our watermarking algorithm and the initial codon distribution of the host genome (y-axis). The watermarking algorithm generates a signature indistinguishable from the rest of the genome. The x-axis here is 100 randomly generated binary signatures of length 1000 mapped to the codon frequency distribution of the individual codons in E. coli

Therefore, each of the binary triplets is represented in terms of their associated codons according to the number of occurrences. Figure 4 shows the correlation to the overall *E. coli* genome codon frequencies that is obtained when an arbitrarily chosen binary signature of length 1000 is represented in terms of DNA nucleotides as determined by our algorithm.

Following design and construction of the entire nucleotide array encompassing the message and algorithm components, there are numerous approaches to incorporate the construct stably in the target organism using standard biotechnological tools for prokaryotes and eukaryotes.

## Conclusions

Our protocol is provably secure in terms of standard cryptanalytic tools, and integrates advanced electronic signature methods with a new watermarking or data-embedding technique, yielding a highly secure and authentication-based product. Importantly, the authentic signature is indistinguishable from random elements in the signature space and the authentication string can be confirmed or denied without disclosing the actual signature. The signature data are not strictly limited in sequence size and may include, but are not limited to, security details, the product production and distribution chain and company licensing details. The resultant data are used as input to establish the security signature in binary according to an algorithm that yields a corresponding string with a nucleotide distribution that closely reflects the natural codon bias of the given host genome. The resulting sequence construct may be inserted into the GMO genome using established genetic engineering technologies such that it is stably inherited through generations. Further increasing the security level can be accomplished by inserting the authentic signature into a subset of the GMO population with the remaining population containing imitation signatures. Authentic signature clones may be identified via PCR by the legitimate owner of the signature or by a designated judge. Importantly, the signature key allowing decoding and authentication is not revealed during this process, thus allowing continued utilization of the key. This provides an increased level of security against whole-genome sequencing and alignment that might increase the probability of identifying a security signature with other standard watermarking approaches.
